# Explainable machine learning model for predicting internal mammary node metastasis in breast cancer: Multi-method development and cross-cohort validation

**DOI:** 10.1016/j.breast.2025.104517

**Published:** 2025-06-09

**Authors:** Yirong Xiang, Jian Tie, Siyuan Zhang, Chen Shi, Changkuo Guo, Yushuo Peng, Zhaoqing Fan, Weihu Wang

**Affiliations:** a Key Laboratory of Carcinogenesis and Translational Research (Ministry of Education/Beijing), Department of Radiation Oncology, Peking University Cancer Hospital and Institute, China; b Breast Center, Peking University Cancer Hospital and Institute, China

**Keywords:** Breast cancer, Internal mammary lymph node metastasis, Machine learning, SHAP

## Abstract

**Background:**

This study developed an explainable machine learning model for baseline internal mammary lymph node metastasis (IMNM) in breast cancer patients.

**Materials and methods:**

This study included three cohorts: a derivation cohort (n = 1997) from Peking University Cancer Hospital, a temporal testing cohort (n = 633) from the same center, and a SEER cohort (n = 51,420). Multiple machine learning strategies were conducted: Least Absolute Shrinkage and Selection Operator (LASSO), Boruta, backward stepwise regression, and best subset for feature selection, and logistic regression (LR), support vector machines (SVM), k-nearest neighbors (KNN), and extreme gradient boosting (XGBoost) for model construction. The best-performing model was validated across internal and temporal testing cohorts. Shapley Additive Explanations (SHAP) analysis was conducted to improve interpretability.

**Results:**

Six clinical features (clinical N stage, size, stage, classification, grade and location) were used to construct the final predictive model with SVM. The model achieved robust performance, with AUCs of 0·811 (0·790–0·843), 0.806 (0·760-0·857) and 0·864 (0·830–0·926) in the training, internal testing and temporal testing cohort, respectively. High-risk patients exhibited significantly worse outcomes with DFS (HR 2·776, 95 % CI: 1·897–4·064, p < 0·001) and OS (HR of 1·962, 95 % CI: 1·853–2·077, p < 0·001). An online prediction tool was established that allows users to input key clinical variables and obtain model-predicted probabilities along with SHAP-based explanations.

**Conclusion:**

This validated and explainable machine learning model offers a practical tool for early risk stratification, aiding clinicians in appropriate baseline imaging selection and adjuvant treatment planning.

## Highlights

• Accurate baseline detection of internal mammary node metastasis (IMNM) is key to staging and treatment in breast cancer.
• IMNM is frequently underdiagnosed in routine clinical practice, especially in patients with neoadjuvant treatment.
• An explainable machine learning model was developed for IMNM prediction using clinical and imaging data.
• A web-based tool was built to support real-world IMNM risk assessment in clinical settings.

## 1 Introduction

Internal mammary lymph node (IMLN) metastases are observed in about 8–27 % of breast cancer cases, particularly in patients with medial or central tumors and axillary node involvement [1]. IMLN metastasis (IMNM) is crucial for tumor staging, prognosis assessing and treatment planning in breast cancer. According to the TNM classification, clinically detected IMNM is categorized as N2b without axillary lymph node involvement and N3b when accompanied by axillary lymph node metastases [2]. Importantly, radiation to the internal mammary lymph chain has been shown to improve prognosis in patients with clinically or pathologically detected IMNM [3,4]. Therefore, careful selection of patients with identified IMNM for adjuvant internal mammary lymph chain radiation is essential to improve therapeutic benefits.

Pathological evaluation, including sentinel lymph node biopsy (SLNB), is not commonly utilized in clinical practice due to variable success rate, technical challenges, invasiveness, and limited impact on survival outcomes [5,6]. Given these limitations, advanced imaging modality including magnetic resonance imaging (MRI) has emerged as a valuable tool for non-invasive assessment of IMNM [7,8]. Additionally, MRI has shown high diagnostic performance in evaluating IMLN metastases. Lee et al. reported that the short axis length of lymph nodes was highly predictive, achieving an AUC of 0.951, with sensitivity and specificity over 90 % at a 4 mm threshold. Kinoshita et al. also demonstrated comparable diagnostic accuracy at a 5 mm threshold. These findings support the critical role of MRI in baseline assessment of IMLN status. Despite its diagnostic value, MRI is not routinely conducted in early-stage breast cancer, leaving a proportion of patients without adequate evaluation of IMLNs. This gap is especially concerning because prior studies have shown that even in axillary node-negative patients, 1.2 %–17.9 % can exhibit IMNM [9]. As a result, missing baseline MRI can lead to undiagnosed IMLN involvement, especially in patients with neoadjuvant therapy with IMNM regression.

Therefore, this study aims to develop and validate a predictive model for IMNM, which can assist clinicians in identifying high-risk patients who would benefit from baseline imaging detection, thereby facilitating precise staging and improving adjuvant radiotherapy planning.

## 2 Materials and methods

### 2.1 Patients

This study included three cohorts of patients with invasive breast cancer. The derivation cohort comprised 1997 patients diagnosed between 2019 and 2021 at Peking University Cancer Hospital. Inclusion criteria were as follows: 1) histologically confirmed invasive breast cancer 2) pre-treatment breast MRI and thoracic CT, and 3) clinical stage M0 at diagnosis. Exclusion criteria included male patients, and those with histological diagnoses of ductal carcinoma in situ (DCIS), lymphoma, or phyllodes tumors. Patients with bilateral, recurrent, occult, or accessory breast cancer were also excluded. The derivation cohort was randomly divided into a training subset (n = 1398) and an internal testing subset (n = 599) in a 7:3 ratio. This split was selected to ensure a sufficient sample size for model training while maintaining an adequate subset for evaluating the model's generalization performance, which aligns with standard practices in machine learning for medical data analysis.

A temporal testing cohort of 633 patients with complete clinical data was recruited between 2022 and 2023 at the same institution for temporal external validation. Additionally, 51,420 patients from the SEER database (2011–2015) were included to detect the prognosis value of the model.

IMNM was defined based on breast MRI and thoracic CT findings as a short-axis diameter ≥5 mm [3,8] and suspicious morphological features such as loss of fatty hilum, high-signal-intensity in diffusion weighted imaging (DWI), or low-signal-intensity in apparent diffusion coefficient map (ADC). IMNM was also defined in cases showing significant reduction in size after neoadjuvant chemotherapy. This study was approved by the Institutional Review Board (IRB) of Peking University Cancer Hospital (2022YJZ100).

### 2.2 Feature selection and model establishment

In data processing, multiple imputation was applied to handle missing data. The Synthetic Minority Oversampling Technique (SMOTE) was implemented in the training cohort to address class imbalance. Restricted cubic splines (RCS) were utilized to assess potential nonlinear relationships in continuous variables, and optimal cut-off points were determined using the least Area Under the Curve (AIU) method.

To identify the most predictive variables, four feature selection methods were applied: Least Absolute Shrinkage and Selection Operator (LASSO), Boruta algorithm, backward stepwise regression, and best subset selection. LASSO regression selected 11 variables based on the λ1se criterion. The Boruta algorithm retained 9 important variables after iterative feature ranking. Both backward stepwise regression and best subset selection identified 6 variables using the Bayesian Information Criterion (BIC) for model simplicity and interpretability.

Subsequently, predictive models were developed using four machine learning algorithms: logistic regression (10.13039/501100009319LR), support vector machines (SVM), k-nearest neighbors (KNN), and extreme gradient boosting (XGBoost). Models were trained using three sets of variables (11, 9, and 6 variables) derived from the above feature selection methods. The final model was selected based on its performance evaluated by the area under the receiver operating characteristic curve (AUC), sensitivity, specificity, balanced accuracy, and recall across both the training and internal testing cohorts.

Model validation was performed using five-fold cross-validation AUCs in the training, internal testing and temporal testing cohort. Calibration curves and decision curve analysis (DCA), adjusted by Platt scaling were employed to assess the reliability and clinical utility of the predictive model. To further assess model robustness, sensitivity analysis was performed using the SEER cohort.

### 2.3 Model explanation and prognostic analysis

Shapley Additive Explanations (SHAP) analysis improved interpretability by quantifying the contribution of each feature to predictions, thereby addressing the black-box nature of machine learning and enhancing model transparency.

A web-based prediction tool was developed using R Shiny, enabling real-time, user-friendly access to the model predictions. The platform allows users to input key clinical variables and obtain individualized risk predictions for IMNM. To enhance interpretability, the tool also integrates SHAP analysis illustrating the contribution of each variable to the final prediction. No user data is stored, and all computations occur within the session to ensure privacy and security.

The optimal threshold for model prediction was determined using the Youden Index derived from the ROC in training cohort. Patients were stratified into high- and low-probability IMNM groups. The prognostic value of the model, including its association with disease-free survival (DFS) and overall survival (OS), was evaluated in the derivation and SEER cohorts using Kaplan-Meier survival curves and Cox proportional hazard models.

**Fig. 1:**
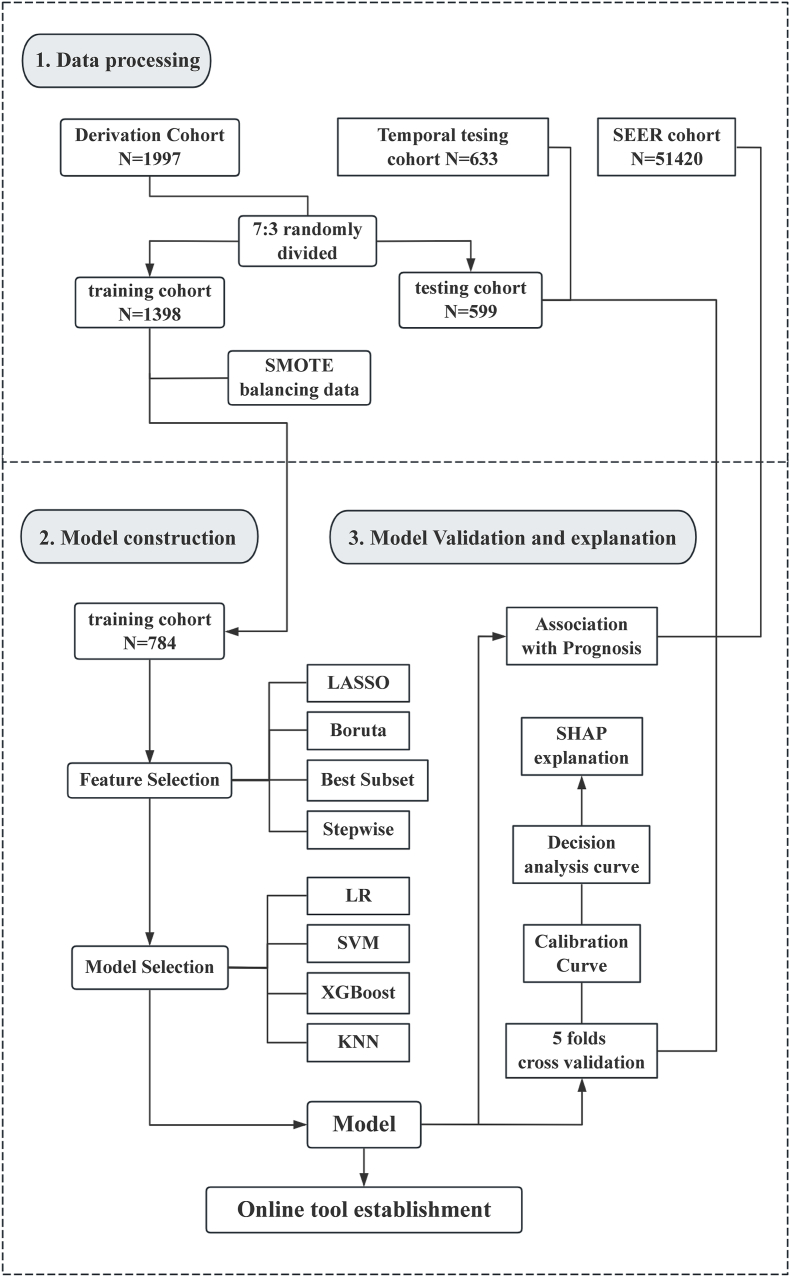
**Workflow of the study.** This figure summarizes the overall study process, including data processing, model construction, and model validation and explanation. Abbreviations: SMOTE, Synthetic Minority Oversampling Technique; SEER, Surveillance, Epidemiology, and End Results; LASSO, Least Absolute Shrinkage and Selection Operator; LR, Logistic Regression; SVM, Support Vector Machine; KNN, k-Nearest Neighbors; XGBoost, Extreme Gradient Boosting; SHAP, Shapley Additive Explanations.

Statistical significance was set at p < 0.05. All analyses were conducted in R software (version4.3.2; https://www.r-project.org), using packages including caret, VIM, foreign, lattice, mice, devtools, DMwR, rms, Boruta, glmnet, autoReg, leaps, xgboost, e1071, dcurves, DALEX, pROC, shiny. The workflow of the study is illustrated in Fig. 1.

## 3 Results

### 3.1 Patient characteristics

The baseline characteristics of the training and testing cohorts are summarized in Table 1 (temporal testing cohort and SEER cohort characteristics are presented in Supplementary Table 1). The mean ages were 50.2 and 50.8 years. Most tumors were classified as cT1 (31.8 % and 37.1 %) or cT2 (55.4 % and 54.1 %). Nodal stages were primarily N0 (49.4 % and 49.1 %) or N1 (46.6 % and 46.4 %). IMNM was identified in 8.0 % of the training cohort and 6.8 % of the testing cohort.

**Table 1:** Baseline characteristics of patients in the training and testing cohorts.

	level	training cohort n = 1398	testing cohort n = 599
age (mean, SD)		50.2 (10.4)	50.8 (10.3)
Clinical T stage	cT1	485 (34.7)	222 (37.1)
	cT2	774 (55.4)	324 (54.1)
	cT3	106 (7.6)	42 (7.0)
	cT4	33 (2.4)	11 (1.8)
Clinical N stage	cN0	691 (49.4)	294 (49.1)
	cN1	651 (46.6)	278 (46.4)
	cN2	29 (2.1)	14 (2.3)
	cN3	27 (1.9)	13 (2.2)
Clinical stage	I	345 (24.7)	149 (24.9)
	II	889 (63.6)	380 (63.4)
	III	164 (11.7)	70 (11.7)
size (mean, SD)		2.95 (2.01)	2.83 (1.80)
ER	Negative	351 (25.1)	122 (20.4)
	Positive	1047 (74.9)	477 (79.6)
PR	Negative	499 (35.7)	199 (33.2)
	Positive	899 (64.3)	400 (66.8)
HER2	Negative	1082 (77.4)	472 (78.8)
	Positive	316 (22.6)	127 (21.2)
classification	Luminal	1088 (77.8)	491 (82.0)
	HER-2 enriched	135 (9.7)	43 (7.2)
	TNBC	175 (12.5)	65 (10.9)
grade	Low	113 (8.6)	48 (8.5)
	Intermediate	892 (67.8)	397 (70.1)
	High	311 (23.6)	121 (21.4)
subtype	IDC	1248 (89.3)	528 (88.1)
	Others	150 (10.7)	71 (11.9)
side	Left	680 (48.6)	281 (46.9)
	Right	718 (51.4)	318 (53.1)
location	Medial or central	588 (42.1)	279 (46.6)
	Lateral	810 (57.9)	320 (53.4)
IMNM	Negative	1286 (92.0)	558 (93.2)
	Positive	112 (8.0)	41 (6.8)

Abbreviations: SD, standard deviation; ER, estrogen receptor; PR, progesterone receptor; HER2, human epidermal growth factor receptor 2; TNBC, triple-negative breast cancer; IDC, invasive ductal carcinoma; IMNM, internal mammary lymph node metastasis.

### 3.2 Feature selection and model construction

The training cohort was balanced using SMOTE, adjusting the class distribution from 112:1286 to approximately 1:1 (Supplementary Table 2). RCS analysis revealed nonlinear relationships between tumor size, age with IMNM, identifying cut-off values of 2.5 cm for tumor size and 50 years for age (Supplementary Fig. 1).

Feature selection using LASSO, Boruta, backward stepwise regression, and best subset selection identified eleven, nine and six key clinical variables for model development (Supplementary Fig. 2 and Supplementary Table 3, with a summary in Supplementary Table 4). Predictive models were constructed using LR, SVM, KNN, and XGBoost algorithms. The SVM model with six selected variables achieved optimal performance and was chosen as the final model. Detailed performance comparisons of different variable sets and algorithms are provided in Table 2.

**Table 2:** Performance of machine learning models (LR, SVM, XGBoost, and KNN) constructed using 6, 9, and 11 selected variables.

Feature	Model	AUC		Sensitivity		Specificity		Balanced Accuracy		Recall	
		training	testing	training	testing	training	testing	training	testing	training	testing
6 variables	LR	0.833	0.790	0.854	0.439	0.679	0.889	0.766	0.664	0.854	0.439
	SVM	0.813	0.805	0.750	0.707	0.779	0.769	0.765	0.738	0.750	0.707
	XGBoost	0.866	0.794	0.738	0.585	0.819	0.760	0.779	0.673	0.738	0.585
	KNN	0.841	0.762	0.580	0.634	0.871	0.663	0.725	0.649	0.580	0.634
9 variables	LR	0.841	0.779	0.774	0.341	0.792	0.898	0.783	0.620	0773	0.341
	SVM	0.824	0.781	0.777	0.707	0.799	0.774	0.788	0.741	0.776	0.707
	XGBoost	0.915	0.782	0.783	0.585	0.895	0.792	0.839	0.689	0.782	0.585
	KNN	0.879	0.770	0.598	0.683	0.906	0.747	0.752	0.715	0.598	0.682
11 variables	LR	0.844	0.974	0.875	0.415	0.676	0.878	0.776	0.646	0.875	0.414
	SVM	0.837	0.784	0.801	0.707	0.770	0.740	0.785	0.724	0.800	0.707
	XGBoost	0.919	0.777	0.795	0.561	0.891	0.801	0.843	0.681	0.794	0.560
	KNN	0.862	0.746	0.619	0.610	0.893	0.746	0.756	0.678	0.619	0.609

Abbreviations: LR, logistic regression; SVM, support vector machines; XGBoost, extreme gradient boosting; KNN, k-nearest neighbors.

**Fig. 2:**
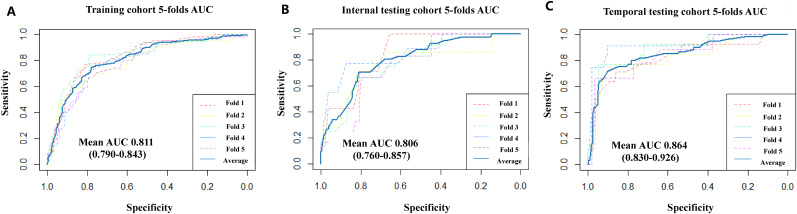
5-Fold Cross-Validation ROC Curves for the training cohort, internal testing cohort and temporal testing cohort.

The predictive performance of the final model was assessed using 5-fold cross-validation ROC curves. The mean AUCs were 0.811 (0.790–0.843) in the training cohort, 0.806 (0.760–0.857) in the internal testing cohort and 0.864 (0.830–0.926) in the temporal testing cohort (Fig. 2). Calibration curves and DCA results was presented in Supplementary Fig. 3. To assess the generalizability of the model, we performed sensitivity analysis using the SEER database (Supplementary Table 5 and Supplementary Fig. 4), showing mean AUCs 0.950 (0.931–0.974).

### 3.3 Model interpretation and prognostic relevance

**Fig. 3:**
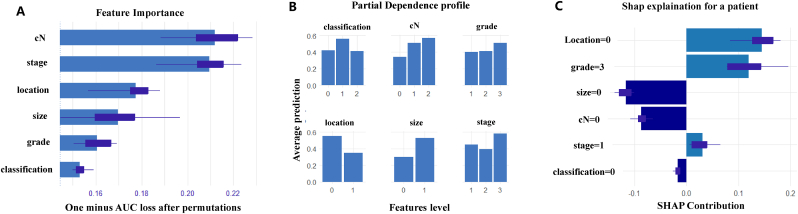
**SHAP Analysis**. (A) Feature importance plot showing the contribution of each variable to the model's performance, measured by one minus AUC loss after permutation, with clinical N stage, tumor stage, and location identified as the top contributors. (B) Partial dependence plots illustrate the relationships between selected features and the predicted probability of IMNM. For classification, levels are defined as 0 = luminal, 1 = HER-2 enriched, and 2 = triple-negative breast cancer; for cN stage, 0 = cN0, 1 = cN1, and 2 = cN2-3. Tumor grade is categorized as 1 = low, 2 = intermediate, and 3 = high, while location is grouped as 0 = medial/central region and 1 = lateral. Tumor size is represented as 0 with ≤2.5 cm and 1 with >2.5 cm, while tumor stage corresponds to I, II, and III. The y-axis indicates the average predicted probability of IMNM. (C) The individual-level SHAP explanation plot demonstrates how each feature contributes to a specific prediction, with the direction and magnitude of contributions visualized for a single patient.

SHAP analysis was used to interpret the final model's predictions. Feature importance (Fig. 3. A) showed that clinical N stage, tumor stage, and location were the top contributors to the model's performance. Partial dependence plots (Fig. 3B) illustrated the relationships between selected features and the model's predictions. For instance, the HER-2 enriched classification showed a higher likelihood of IMNM. An individual-level SHAP explanation (Fig. 3C) further illustrated the contribution of each feature to a specific prediction outcome. A web-based prediction tool successfully provides individualized risk predictions and SHAP-based explanations.This online tool can be accessed at https://bcprediction.shinyapps.io/imnmprediction/.

**Fig. 4:**
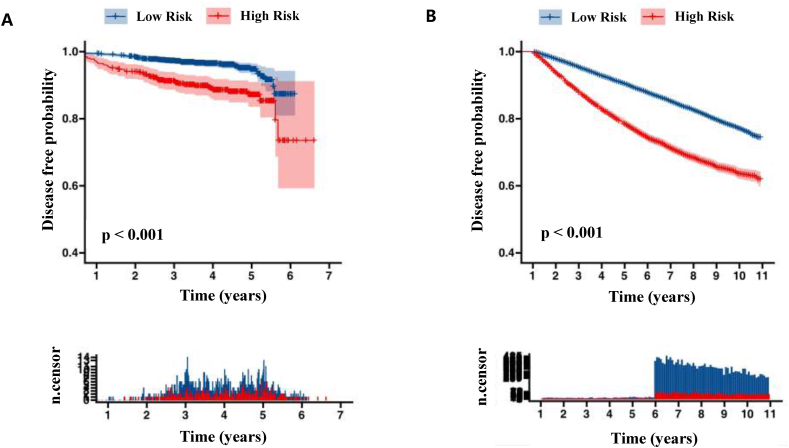
**Kaplan-Meier Curves for Predicted High- and Low-Risk Groups** (A) disease-free survival (DFS) in the derivation cohort, comparing predicted high-risk (red) and low-risk (blue) groups. (B) overall survival (OS) in the SEER cohort, comparing predicted high-risk (red) and low-risk (blue) groups.

In the derivation cohort (median follow-up: 3.89 years), DFS was significantly lower in the high-risk group predicted by the model compared to the low-risk group (HR = 2.776, 95 % CI: 1.897–4.064, p < 0.001), with a 5-year DFS of 87.2 % vs 94.7 % (Fig. 4A). Similarly, in the SEER cohort (median follow-up: 11 years), OS was worse in the high-risk group (HR = 1.962, 95 % CI: 1.853–2.077, p < 0.001), with a 5-year OS of 78.5 % vs 90.4 % (Fig. 4B). For IMNM positive patients in both cohorts, survival outcomes were poorer compared to IMNM negative patients, with detailed results shown in Supplementary Fig. 5.

## 4 Discussion

This study developed and validated a predictive model for internal mammary lymph node metastasis in breast cancer patients using various feature selection and model construction methods. The model demonstrated robust predictive performance with AUCs of 0.806–0.864 in the training, internal testing and temporal testing cohorts. High-risk patients identified by the model had significantly worse prognosis compared to low-risk patients. The study also incorporated SHAP analysis to enhance model interpretability. An online tool was established for clinical use.

Historically, internal mammary lymph node management in breast cancer involved invasive procedures such as surgical dissection and sentinel lymph node biopsy. However, studies have demonstrated that these approaches do not improve survival outcomes and carry risks associated with their invasiveness [6,10]. As a result, clinical practice has shifted towards non-invasive imaging evaluation combined with postoperative radiotherapy when appropriate [8,9,11,12]. Despite its critical role, imaging modalities like MRI are not routinely performed in guidelines for all breast cancer patients, leading to underdiagnosis of high-risk individuals. This issue is particularly prominent in patients undergoing neoadjuvant chemotherapy, where treatment can obscure the imaging visibility of metastases in baseline [13,14]. Accurate diagnosis of IMNM is essential not only for staging but also for guiding treatment strategies, particularly adjuvant radiotherapy. Previous trials have demonstrated that regional nodal irradiation, including internal mammary node irradiation (IMNI), can improve the prognosis of early-stage breast cancer [[15], [16], [17]]. However, IMNI increases dose-volume of heart and lungs which relates to radiation-induced cardiotoxicity and pulmonary complications [18]. Therefore, careful patient selection for IMNI is critical, emphasizing the need for accurate pre-treatment evaluation. This study addresses this clinical gap by developing a model to help surgical oncologists identify high-risk patients for baseline imaging and to guide radiation oncologists in planning internal mammary irradiation, even when baseline imaging is unavailable.

Previous studies have identified several risk factors associated with internal mammary lymph node metastasis, including larger tumor size [19,20], advanced axillary lymph node stage [19,21], medial or central tumor location [[20], [21], [22]], younger age [19], and certain molecular subtypes such as HER-2 enriched breast cancer [20,23,24]. However, these studies primarily focused on isolated risk factors without integrating multiple clinical variables into a comprehensive predictive model. To address this limitation, we developed and validated a robust predictive machine learning model for IMNM prediction. In the present study, we selected six clinical features including cN (nodal status), stage, size, classification, grade, and location. These predictive variables not only align with previously reported risk factors but also reflect clinically observed patterns across different stages of breast cancer. Internal mammary chain metastasis risk is dependent on the factors mentioned (localization and stage of primary tumor), which are not directly related nor the same as for axillary involvement risk. Detection is more complicated than axillary nodes, which are studied in various models [25,26]. Seen the higher risk in more advanced disease, training of the model for both early stage (T1-2) and more advanced stage (T3-4) would be helpful to better understand risk in the early group. In locally advanced breast cancer, tumor burden—such as nodal status (cN), tumor size, and overall clinical stage—is a major determinant of both the likelihood of IMNM and the potential benefit of internal mammary irradiation. However, in earlier-stage patients (such as those predominantly included in our study), where the tumor burden is relatively lower, the propensity for internal mammary spread may be more closely related to tumor-intrinsic characteristics. For example, tumors located in the medial or central quadrants are more likely to exhibit lymphatic drainage through the internal mammary chain, and biological features such as HER2-enriched subtype and poor differentiation are associated with more aggressive behavior and higher metastatic potential to the internal mammary region. These insights emphasize the multifactorial nature of IMNM risk and justify the inclusion of both tumor burden and tumor biological variables in the predictive model. SHAP analysis offered further insights by quantifying the individual contributions of each feature to model predictions. For example, HER-2 enriched subtype showed a higher likelihood of internal mammary node involvement compared to luminal and triple-negative subtypes, which is consistent with previous studies reporting its aggressive metastatic nature to the internal mammary lymph node chain [20,23]. By enhancing model interpretability, SHAP provides actionable insights to support risk stratification and clinical decision-making.

Geographic variation in breast cancer presentation and prognosis has been well documented in global cancer statistics. Asian women tend to be diagnosed at a younger age and exhibit more aggressive subtypes (e.g., HER2-positive, triple-negative), along with lower rates of early-stage detection, due to factors such as breast density, limited screening access, and healthcare disparities [[27], [28], [29], [30]]. These differences were also evident in our study cohort, where the average mean age of the cohort is low, which can reflect a poorer prognosis overall, as well as regional difference of early breast cancer presentation in Asia compared to the SEER database used. This difference in cohort can introduce bias in the model. Retraining with cohorts of other geographical cohorts will help the predictive precision of the tool proposed. Nevertheless, our model demonstrated generalizability when externally validated in the SEER cohort, with comparable predictive accuracy and consistent risk factor associations. This suggests that the model captures biologically relevant patterns that are applicable across geographically and ethnically diverse populations. Retraining with other regional cohorts may still further improve precision, but these findings support the model's robustness in real-world settings. These considerations emphasize the importance of developing population-specific prediction tools and reinforce the rationale for validating them across different geographic contexts.

This study offers several strengths. First, SHAP analysis provides clear insights into feature contributions, making the model transparent and explainable. Also, an interactive web-based prediction tool (https://bcprediction.shinyapps.io/imnmprediction/) that allows clinicians and researchers to easily access model-predicted probabilities and SHAP-based explanations. This tool enhances the interpretability and accessibility of the model, facilitating its integration into clinical decision-making. Second, the selected clinical features—cN (nodal status), stage, size, classification, grade, and location—are widely used, easily accessible, and minimally controversial in clinical practice, supporting the model's practicality. This supports real-world applicability, allowing clinicians to easily apply the model using standard clinical variables without requiring additional costly or invasive tests. Third, the integration of multiple feature selection methods (LASSO, Boruta, best subset, and stepwise regression) and machine learning algorithms (logistic regression, SVM, XGBoost, and KNN) enhances its predictive power and adaptability. This integrative approach not only improves predictive accuracy but also provides flexibility for implementation across different clinical settings and patient populations. Finally, the large sample size and comprehensive validation across multiple cohorts, including internal and temporal testing cohorts, ensure the model's robustness and generalizability.

This study also has some limitations. First, the diagnosis of internal mammary lymph node metastasis relied on imaging modalities such as MRI and CT, which, in spite of widely used and non-invasive, may introduce variability compared to the pathological examinations. Second, the relatively short follow-up limits the assessment of long-term outcomes. Third, the single-center nature of the derivation cohort may limit generalizability, despite validation across temporal and external cohorts. Also, the SEER cohort demonstrated a relatively low incidence of IMNM, which may have led to an overestimated model performance. Nevertheless, the SEER analysis serves as an external validation demonstrating the model's applicability to a broader population, albeit with caution. Future research should focus on multi-center validation with longer follow-up periods to enhance the model's robustness and clinical utility. Another limitation is that the model was developed in an Asian population, which may raise concerns about generalizability. Nevertheless, consistent IMNM risk factors were identified in the SEER cohort (Supplementary Table 5), suggesting that the model captures biologically meaningful patterns applicable to diverse populations. Nevertheless, despite these limitations, the model provides valuable decision support for patients who may otherwise be under-evaluated for IMNM risk, particularly in resource-constrained settings.

## 5 Conclusion

This study developed and validated a predictive model for internal mammary node metastasis in breast cancer using baseline clinical features. The model demonstrated strong robustness and clinical utility, aiding in patient stratification and treatment planning.

## CRediT authorship contribution statement

**Yirong Xiang:** Writing – review & editing, Writing – original draft, Validation, Methodology, Investigation, Formal analysis, Data curation, Conceptualization. **Jian Tie:** Writing – review & editing, Visualization, Supervision, Methodology, Investigation, Data curation, Conceptualization. **Siyuan Zhang:** Writing – review & editing, Validation, Supervision, Resources, Investigation, Data curation. **Chen Shi:** Supervision, Resources, Project administration, Methodology, Investigation, Data curation. **Changkuo Guo:** Writing – review & editing, Resources, Methodology, Formal analysis, Data curation, Conceptualization. **Yushuo Peng:** Visualization, Methodology, Investigation, Data curation. **Zhaoqing Fan:** Visualization, Supervision, Resources, Data curation. **Weihu Wang:** Supervision, Funding acquisition, Conceptualization.

## Data sharing statement

The datasets used during the current study are available from the corresponding author upon reasonable request.

## Ethical approval

This study was approved by the Institutional Review Board (IRB) of Peking University Cancer Hospital (2022YJZ100).

## Funding

This work was supported by the Beijing Hospitals Authority's Ascent Plan, Grant No. DFL20220902.

## Declaration of interests

The authors declare no competing interests.
