# Supplementary material for: Explainable machine learning model for predicting internal mammary node metastasis in breast cancer: Multi-method development and cross-cohort validation
Source: Breast. 2025 Jun 9;82:104517. doi: 10.1016/j.breast.2025.104517 (PMC12205679; doi:10.1016/j.breast.2025.104517)
Supplement: Multimedia component 1 [file mmc1.docx]

**Supplementary data Outlines**

**Supplementary Table 1**. Baseline Characteristics of Patients in the Temporal Testing and SEER Cohorts.

**Supplementary Table 2**. Characteristics of IMNM Samples Before and After SMOTE Balancing.

**Supplementary Figure 1**. Nonlinear Relationships Identified by Restricted Cubic Splines (RCS) between age, tumor size and Internal mammary lymph node metastasis (IMNM).

**Supplementary Figure 2**. Results of Feature Selection.

**Supplementary Table 3**. Stepwise Regression Results with univariable, multivariable and final stepwise regression.

**Supplementary Table 4**. Summary of Selected Variables by Feature Selection Methods.

**Supplementary Figure 3**. Calibration and Decision Curve Analysis.

**Supplementary Table 5**. Distribution of Clinical and Pathological Features by IMNM Status in the SEER Cohort and Their Univariate and Multivariate Associations

**Supplementary Figure 4.** 5-Fold Cross-Validation ROC Curves, Calibration and Decision Curve Analysis in SEER cohort.

**Supplementary Figure 5**. Kaplan-Meier Curves for internal mammary lymph node metastasis (IMNM) positive and negative groups.

**Supplementary Table 1. Baseline Characteristics of Patients in the Temporal Testing and SEER Cohorts**

|  | level | temporal testing cohort  n=633 | SEER cohort  N=51420 |
| --- | --- | --- | --- |
| age (mean, SD) | | 50.21 (10.42) | 50.69 (10.40) |
| Clinical T stage | cT1 | 201 (31.8) | 33666 (65.5) |
|  | cT2 | 361 (57.0) | 14654 (28.5) |
|  | cT3 | 51 (8.1) | 2306 (4.5) |
|  | cT4 | 20 (3.2) | 794 (1.5) |
| Clinical N stage | cN0 | 282 (44.5) | 38283 (74.5) |
|  | cN1 | 260 (41.1) | 10400 (20.2) |
|  | cN2 | 43 (6.8) | 1828 (3.6) |
|  | cN3 | 48 (7.6) | 909 (1.8) |
| Clinical stage | I | 153 (24.2) | 29895 (58.1) |
|  | II | 361 (57.0) | 17267 (33.6) |
|  | III | 119 (18.8) | 4258 (8.3) |
| size (mean, SD) | | 2.95 (2.01) | 3.05 (1.87) |
| ER | Negative | 141 (22.3) | 7810 (15.2) |
|  | Positive | 492 (77.7) | 43610 (84.8) |
| PR | Negative | 150 (23.7) | 13228 (25.7) |
|  | Positive | 483 (76.3) | 38192 (74.3) |
| HER2 | Negative | 473 (74.7) | 44653 (86.8) |
|  | Positive | 160 (25.3) | 6767 (13.2) |
| classification | Luminal | 516 (81.5) | 44087 (85.7) |
|  | HER-2 enriched | 53 (8.4) | 1912 (3.7) |
|  | TNBC | 64 (10.1) | 5421 (10.5) |
| grade | Low | 45 (7.1) | 14146 (27.5) |
|  | Intermediate | 444 (70.1) | 22524 (43.8) |
|  | High | 144 (22.7) | 14750 (28.7) |
| subtype | IDC | 596 (94.2) | 43854 (85.3) |
|  | Others | 37 (5.8) | 7566 (14.7) |
| side | Left | 331 (52.3) | 26021 (50.6) |
|  | Right | 302 (47.7) | 25398 (49.4) |
| location | Medial or central | 269 (42.5) | 18556 (36.1) |
|  | Lateral | 364 (57.5) | 32864 (63.9) |
| IMNM | Negative | 572 (90.4) | 51228 (99.6) |
|  | Positive | 61 (9.6) | 192 (0.4) |

Abbreviations: SD, standard deviation; ER, estrogen receptor; PR, progesterone receptor; HER2, human epidermal growth factor receptor 2; TNBC, triple-negative breast cancer; IDC, invasive ductal carcinoma; IMNM, internal mammary lymph node metastasis.

**Supplementary Table 2. Characteristics of IMNM Samples Before and After SMOTE Balancing**

|  | level | Before smote  n=112 | After smote  n=336 | P value |
| --- | --- | --- | --- | --- |
| age (mean, SD) | | 49.00 (11.19) | 48.61 (9.94) | 0.727 |
| Clinical T stage | cT1 | 14 (12.5) | 50 (14.9) | 0.573 |
|  | cT2 | 66 (58.9) | 174 (51.8) |  |
|  | cT3 | 24 (21.4) | 89 (26.5) |  |
|  | cT4 | 8 (7.1) | 23 (6.8) |  |
| Clinical N stage | cN0 | 26 (23.2) | 76 (22.6) | 0.986 |
|  | cN1 | 69 (61.6) | 210 (62.5) |  |
|  | cN2-3 | 17 (15.2) | 50 (14.9) |  |
| Clinical stage | I | 5 (4.5) | 19 (5.7) | 0.536 |
|  | II | 68 (60.7) | 184 (54.8) |  |
|  | III | 39 (34.8) | 133 (39.6) |  |
| size (mean, SD) | | 4.80 (3.42) | 4.86 (2.91) | 0.849 |
| ER | Negative | 44 (39.3) | 155 (46.1) | 0.249 |
|  | Positive | 68 (60.7) | 181 (53.9) |  |
| PR | Negative | 53 (47.3) | 172 (51.2) | 0.548 |
|  | Positive | 59 (52.7) | 164 (48.8) |  |
| HER2 | Negative | 68 (60.7) | 192 (57.1) | 0.580 |
|  | Positive | 44 (39.3) | 144 (42.9) |  |
| classification | Luminal | 75 (67.0) | 192 (57.1) | 0.172 |
|  | HER-2 enriched | 22 (19.6) | 91 (27.1) |  |
|  | TNBC | 15 (13.4) | 53 (15.8) |  |
| grade | Low | 2 (1.8) | 6 (1.8) | 0.387 |
|  | Intermediate | 64 (57.1) | 167 (49.7) |  |
|  | High | 46 (41.1) | 163 (48.5) |  |
| subtype | IDC | 99 (88.4) | 284 (84.5) | 0.394 |
|  | Others | 13 (11.6) | 52 (15.5) |  |
| side | Left | 62 (55.4) | 197 (58.6) | 0.619 |
|  | Right | 50 (44.6) | 139 (41.4) |  |
| location | Medial or central | 79 (70.5) | 217 (64.6) | 0.300 |
|  | Lateral | 33 (29.5) | 119 (35.4) |  |

SMOTE, Synthetic Minority Oversampling Technique; SD, standard deviation; ER, estrogen receptor; PR, progesterone receptor; HER2, human epidermal growth factor receptor 2; TNBC, triple-negative breast cancer; IDC, invasive ductal carcinoma; IMNM, internal mammary lymph node metastasis.


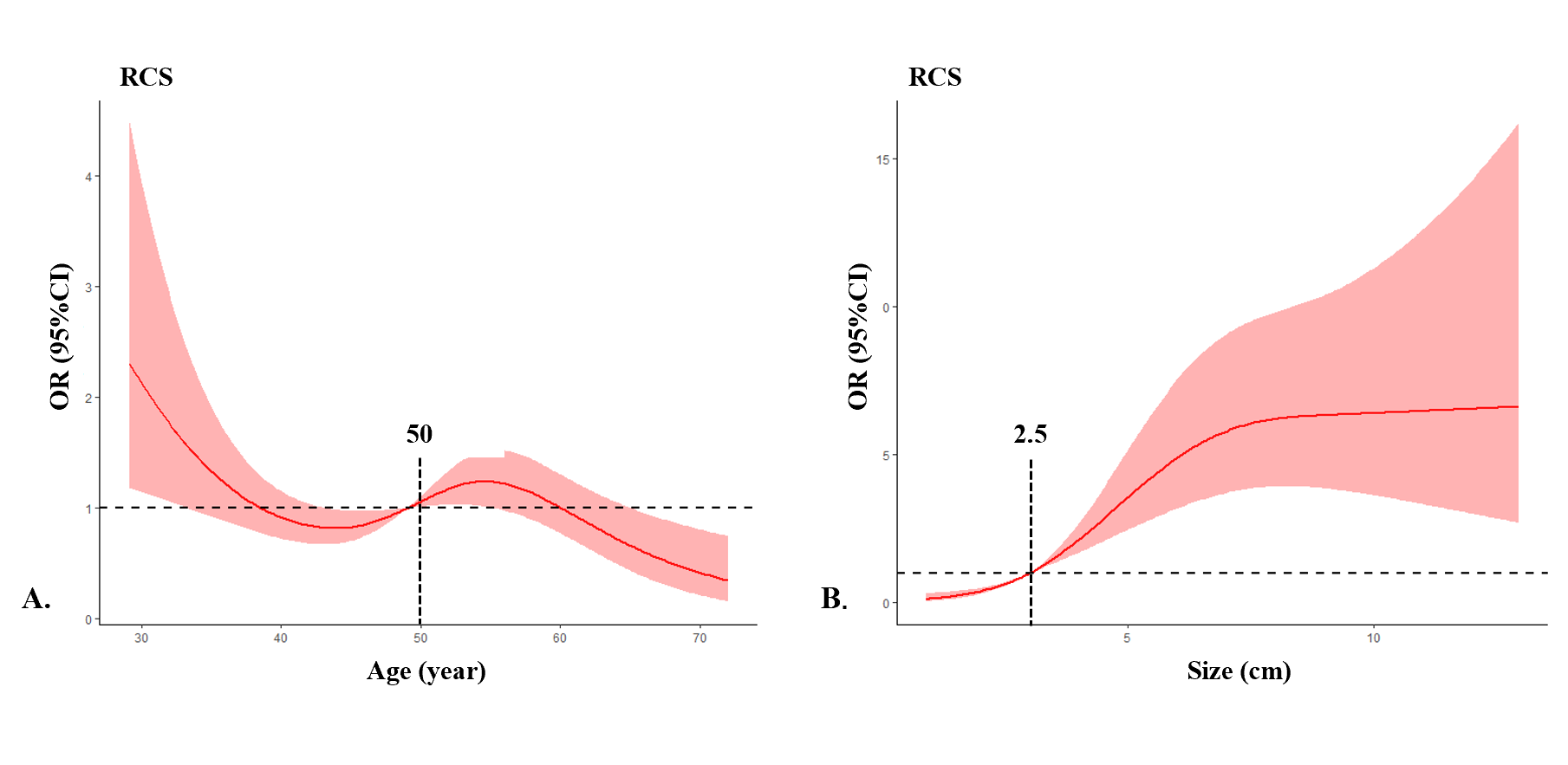


**Supplementary Figure 1. Nonlinear Relationships Identified by Restricted Cubic Splines (RCS) between age, tumor size and Internal mammary lymph node metastasis (IMNM).**

(A) Age: Using the Akaike Information Criterion (AIC), 4 nodes were selected as optimal, with a significant p-value for nonlinearity (p = 0.022). The cut-off point for age was identified as 50 years. (B) Tumor Size: 4 nodes were also chosen as optimal, with strong evidence of nonlinearity (p < 0.001). The cut-off point for tumor size was determined to be 2.5 cm. The red lines represent the odds ratio (OR) estimates, and the shaded areas indicate the 95% confidence intervals.


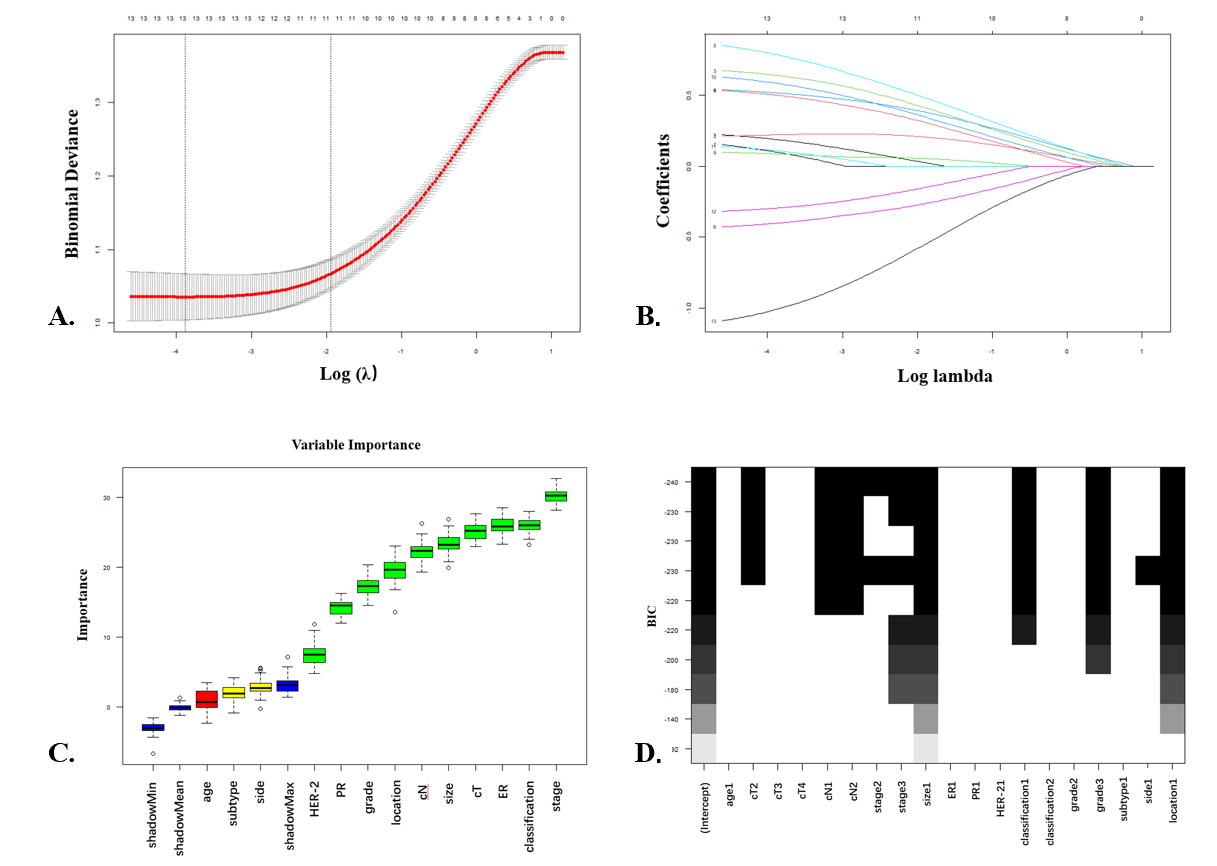


**Supplementary Figure 2. Results of Feature Selection.** (A) LASSO Regression Tenfold cross-validation plot showing the relationship between binomial deviance and log(λ). The two dotted lines represent λmin and λ1se, with the optimal λ determined at λ1se. B) LASSO coefficient plot. Each line corresponds to a clinical variable. As the penalty parameter λ increases, the coefficients gradually shrink toward zero, with nonzero coefficients representing selected variables. (C) Boruta: Based on importance scores, 9 variables with significance (green color) and importance scores >2 were selected for inclusion. (D) Best Subset Selection: The Bayesian Information Criterion (BIC) was used to determine the optimal model. The uppermost line with the lowest BIC value indicates the final set of selected variables.

**Supplementary Table 3. Stepwise Regression Results with univariable, multivariable and final stepwise regression.**

|  |  | **OR (univariable)** | **OR (multivariable)** | **OR (final)** |
| --- | --- | --- | --- | --- |
| **age** | ≤50 | 1.06 (0.80-1.41, p=.679) |  |  |
|  | >50 |  |  |  |
| **Clinical T stage** | cT1 |  |  |  |
|  | cT2 | 2.16 (1.49-3.14, p<.001) | 0.50 (0.26-0.97, p=.041) | 0.48 (0.25-0.93, p=.030) |
|  | cT3 | 10.82 (6.30-18.57, p<.001) | 1.19 (0.51-2.79, p=.687) | 1.17 (0.50-2.75, p=.712) |
|  | cT4 | 7.27 (3.24-16.30, p<.001) | 1.21 (0.40-3.70, p=.737) | 1.22 (0.40-3.72, p=.729) |
| **Clinical N stage** | cN0 |  |  |  |
|  | cN1 | 3.34 (2.42-4.61, p<.001) | 1.82 (1.19-2.76, p=.005) | 1.83 (1.20-2.77, p=.005) |
|  | cN2-3 | 12.04 (6.21-23.36, p<.001) | 4.62 (2.00-10.67, p<.001) | 5.06 (2.19-11.65, p<.001) |
| **Clinical stage** | I |  |  |  |
|  | II | 4.22 (2.51-7.08, p<.001) | 2.17 (1.02-4.62, p=.045) | 2.18 (1.02-4.66, p=.044) |
|  | III | 18.57 (10.31-33.43, p<.001) | 3.36 (1.33-8.50, p=.010) | 3.21 (1.27-8.10, p=.013) |
| **Size** | ≤2.5 |  |  |  |
|  | >2.5 | 5.00 (3.59-6.96, p<.001) | 3.35 (2.00-5.61, p<.001) | 3.42 (2.05-5.73, p<.001) |
| **ER** | Negative |  |  |  |
|  | Positive | 0.41 (0.30-0.55, p<.001) |  |  |
| **PR** | Negative |  |  |  |
|  | Positive | 0.63 (0.48-0.84, p=.002) |  |  |
| **HER2** | Negative |  |  |  |
|  | Positive | 2.54 (1.87-3.47, p<.001) |  |  |
| **classification** | Luminal |  |  |  |
|  | HER-2 enriched | 3.94 (2.62-5.93, p<.001) | 2.28 (1.23-4.25, p=.009) | 2.73 (1.65-4.49, p<.001) |
|  | TNBC | 1.43 (0.95-2.13, p=.084) | 1.21 (0.65-2.24, p=.552) | 1.15 (0.70-1.88, p=.589) |
| **grade** | Low |  |  |  |
|  | Intermediate | 4.43 (1.85-10.59, p<.001) | 2.65 (0.95-7.39, p=.062) | 2.87 (1.04-7.96, p=.043) |
|  | High | 11.06 (4.57-26.77, p<.001) | 5.46 (1.89-15.79, p=.002) | 6.22 (2.18-17.74, p<.001) |
| **subtype** | IDC |  |  |  |
|  | Others | 1.06 (0.71-1.57, p=.773) |  |  |
| **side** | Left |  |  |  |
|  | Right | 0.61 (0.46-0.81, p<.001) | 0.75 (0.53-1.08, p=.122) | 0.75 (0.53-1.07, p=.116) |
| **location** | Medial or central |  |  |  |
|  | Lateral | 0.36 (0.27-0.49, p<.001) | 0.31 (0.22-0.44, p<.001) | 0.31 (0.22-0.44, p<.001) |

**Supplementary Table 4. Summary of Selected Variables by Feature Selection Methods**

| **Feature Selection Method** | **Selected Variables** |
| --- | --- |
| LASSO Regression | age, cT, cN, stage, size, ER, HER2, classification, grade, side, location |
| Backward Stepwise Regression | cN, stage, size, classification, grade, location |
| Boruta Algorithm | cT, cN, stage, size, ER, PR, classification, grade, location |
| Best Subset Selection | cN, stage, size, classification, grade, location |

Abbreviations: cT, clinical T stage; cN, clinical N stage; ER, estrogen receptor; HER2, human epidermal growth factor receptor 2; PR, progesterone receptor; IMNM, internal mammary lymph node metastasis.


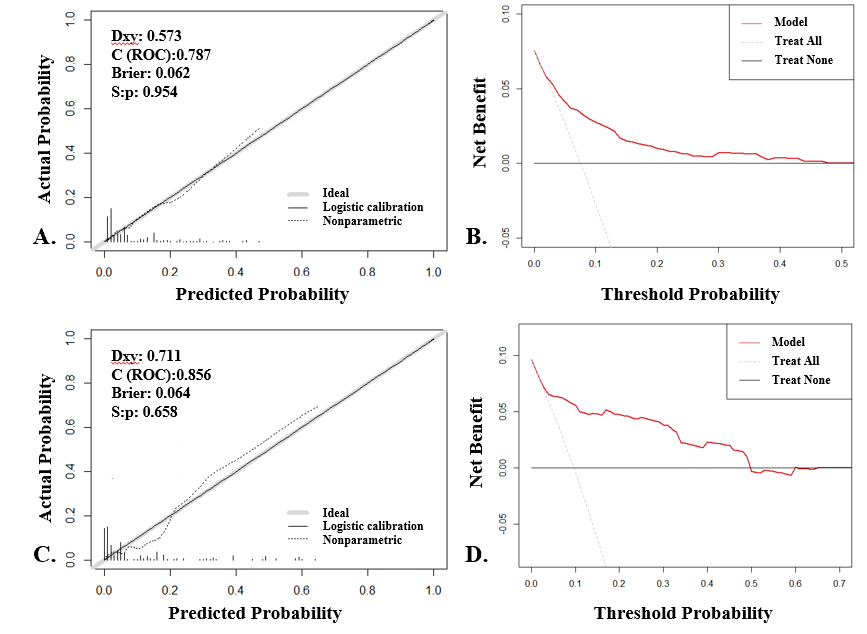


**Supplementary Figure 3. Calibration and Decision Curve Analysis.** (A, C) Calibration curves for the derivation cohort and temporal testing cohort. The x-axis represents the predicted probability, and the y-axis represents the actual probability. The solid line indicates perfect calibration, while the dashed line shows the model’s performance. (B, D) Decision curve analysis (DCA) for the derivation cohort and temporal testing cohort. The x-axis represents the threshold probability, and the y-axis represents the net benefit. The red line indicates the net benefit compared to the "treat all" and "treat none" strategies.

**Supplementary Table 5**. Distribution of Clinical and Pathological Features by IMNM Status in the SEER Cohort and Their Univariate and Multivariate Associations

|  | level | Without IMNM  n=51228 | With IMNM  n=192 | Univariate  *p*-value | Multivariate  *p*-value |
| --- | --- | --- | --- | --- | --- |
| age (years) | ≤50 | 9746 | 37 | 0.931 | 0.685 |
|  | ＞50 | 41482 | 155 |  |  |
| Clinical T stage | cT1 | 33628 | 38 | <0.001 | 0.009 |
|  | cT2 | 14574 | 80 |  |  |
|  | cT3 | 2266 | 40 |  |  |
|  | cT4 | 760 | 34 |  |  |
| Clinical N stage | cN0 | 38283 | 0 | <0.001 | <0.001 |
|  | cN1 | 10383 | 17 |  |  |
|  | cN2 | 1792 | 36 |  |  |
|  | cN3 | 770 | 139 |  |  |
| Clinical stage | I | 29895 | 0 | <0.001 | 0.985 |
|  | II | 17253 | 14 |  |  |
|  | III | 4080 | 178 |  |  |
| ER | Negative | 7737 | 73 | <0.001 | 0.024 |
|  | Positive | 43491 | 119 |  |  |
| PR | Negative | 13129 | 99 | <0.001 | 0.020 |
|  | Positive | 38099 | 93 |  |  |
| HER2 | Negative | 44516 | 137 | <0.001 | 0.003 |
|  | Positive | 6712 | 55 |  |  |
| classification | Luminal | 43962 | 125 | <0.001 | 0.007 |
|  | HER-2 enriched | 1890 | 22 |  |  |
|  | TNBC | 5376 | 45 |  |  |
| grade | Low | 14131 | 15 | <0.001 | 0.227 |
|  | Intermediate | 22461 | 63 |  |  |
|  | High | 14636 | 114 |  |  |
| side | Left | 25918 | 103 | 0.399 | 0.260 |
|  | Right | 25309 | 89 |  |  |
| location | Medial or central | 18465 | 91 | <0.001 | <0.001 |
|  | Lateral | 32763 | 101 |  |  |

**
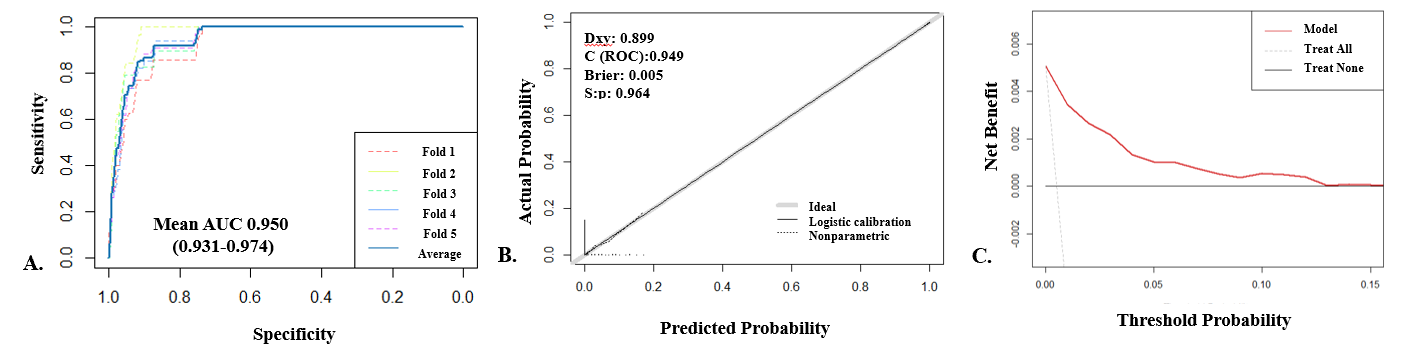
**

**Supplementary Figure 4.** 5-Fold Cross-Validation ROC Curves (A), Calibration (B) and Decision Curve Analysis (C) in SEER cohort. These analyses were performed as part of sensitivity analysis to assess model robustness in an external population.


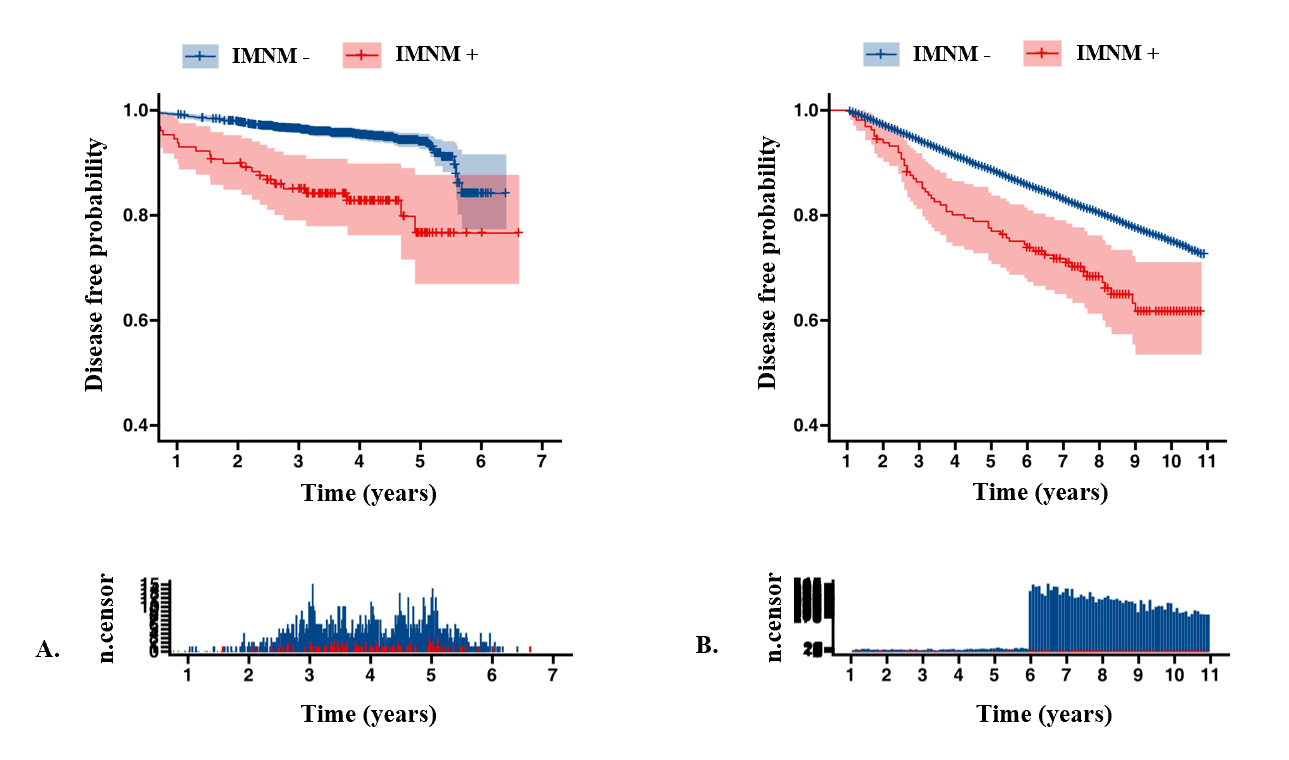


**Supplementary Figure 5. Kaplan-Meier Curves for internal mammary lymph node metastasis (IMNM) positive and negative groups (A)** Disease-free survival (DFS) in the derivation cohort, comparing IMNM+ (red) and IMNM- (blue) groups. (B) Overall survival (OS) in the SEER cohort, comparing IMNM+ (red) and IMNM- (blue) groups.
